# Valuing individual characteristics and the multifunctionality of urban green spaces: The integration of sociotope mapping and hedonic pricing

**DOI:** 10.1371/journal.pone.0212277

**Published:** 2019-03-06

**Authors:** Piotr Czembrowski, Edyta Łaszkiewicz, Jakub Kronenberg, Gustav Engström, Erik Andersson

**Affiliations:** 1 Faculty of Economics and Sociology, University of Lodz, Lodz, Poland; 2 The Beijer Institute, The Royal Swedish Academy of Sciences, Stockholm, Sweden; 3 Stockholm Resilience Centre, Stockholm University, Stockholm, Sweden; Irstea, FRANCE

## Abstract

We categorize Stockholm’s urban green spaces according to the use values and social meanings they support, based on a sociotope mapping, and estimate their impact on property prices with a hedonic pricing model. The approach allows us to identify the most and least desired green space characteristics (attributes) and to assess the willingness to pay for the multifunctionality of green spaces. To do this, we test the following hypotheses, each with a separate hedonic pricing model:

- the proximity of all green space characteristics increases the property prices, but the specific monetary value of these characteristics differs;
- the multifunctionality of green spaces is well recognized and highly valued by real estate buyers.

We find partial support for the first hypothesis: the green space attributes of “aesthetics”, “social activity” and “nature” seem to be desired by real estate buyers, whereas “physical activity” and “play” seem not to be desired. We also find support for the second hypothesis: the higher the number of characteristics an urban green space has, the stronger its impact on property prices. This study furthers the discussion on the economic value of urban green spaces by assigning monetary value to their perceived character and use values. In doing so, it highlights the need to understand green spaces both as ecological features and social constructs.

## Introduction

After years of research on the benefits that urban dwellers derive from green spaces, all stakeholders should already be well aware that green infrastructure is a crucial part of the urban tissue [1,2]. However, to date, studies most often focus on individual benefits or ecosystem services, which leaves space for interdisciplinary and pluralistic research and planning approaches [3]. With the recent interest in nature-based solutions and renaturing cities, increasing attention has been paid to the multifunctionality of urban green infrastructure–in terms of both the multiple benefits and the services that it provides [4,5] and the multiple ways inhabitants use urban green spaces [6].

The multifunctionality of urban green spaces can be addressed within the now increasingly popular monetary valuation framework. A pluralistic approach here would be to evaluate urban green spaces based on the different functions, benefits or services that they provide, and then estimate the monetary value of these green spaces (or at least of the said characteristics). The most typical monetary valuation method for studying green spaces based on their different characteristics would be a choice experiment, which captures people’s stated preferences regarding the different characteristics based on hypothetical scenarios (for relevant examples, see, e.g., [7,8]). We propose an alternative approach–valuing multifunctionality with the use of hedonic pricing, a method based on revealed preferences observed primarily in the real estate market. Our approach builds on the recent applications of hedonic pricing to study the different characteristics of environmental amenities, captured by the use of non-monetary valuation methods such as participatory GIS (the method of capturing spatially explicit opinions, experiences and values expressed by respondents in the so-called geo-questionnaires) [9,10]. If designed properly and with sufficient data on green spaces and their characteristics, we argue that hedonic pricing can provide a powerful analytical lens. However, one has to bear in mind the inevitable limitation of the hedonic pricing method–that it assumes complete recognition of the characteristics in the model by real estate buyers [11].

In this study, we used hedonic pricing to estimate the monetary value of various social meanings and use values of green spaces recognized through sociotope mapping. A sociotope is a defined space able to provide distinct social functions (as compared to a biotope serving biological functions). Such a mapping has been put forth in Stockholm, Sweden, to reflect expert knowledge on the characteristics of green spaces, e.g., whether a green space is equipped with a playground or characterized by the abundance of flowers [12]. The mapping is based on expert assessment and user surveys (questionnaires and interviews). It also employs the classification of public spaces into four broad categories–larger public spaces (e.g. larger recreational spaces), distinct bounded units (e.g. squares and parks), built-up areas (e.g. yards and sidewalks) and transportation spaces (e.g. harbors). However, in our integration of sociotope mapping and hedonic pricing, we did not use this distinction of green spaces as it is not in line with the goal of this study. The integration of sociotope mapping and hedonic pricing should provide unique knowledge of the marginal willingness to pay for the different characteristics of urban green spaces by real estate buyers in Stockholm.

Our hedonic pricing study consisted of two stages, each with its own goal and hypothesis. In the first stage, our aim was to create a ranking of the selected five general characteristics of urban green spaces (i.e. categories) according to their monetary value. Our hypothesis was that the proximity of all green space characteristics increases property prices but that the individual economic value of these characteristics differs. The second part of the study was designed to estimate the monetary value of the multifunctionality of green spaces. Our hypothesis was that the multifunctionality of green spaces is well recognized and highly valued by real estate buyers.

The article is organized as follows. In the following section, we describe Stockholm as the study site, sketch the broader context of our hedonic pricing in the existing literature, describe a method for integrating sociotope mapping with hedonic pricing, list the full set of variables and discuss the technical issues of estimating a hedonic pricing model. The method section is followed by the results, and the article concludes with a discussion and conclusions.

## Methods

### Study area

Urban green spaces in Stockholm, along with the services and benefits that they provide, have already been thoroughly studied, e.g., as one of the local case studies within the Millennium Ecosystem Assessment and the Cities and Biodiversity Outlook, both high-level international initiatives addressing ecosystems and human well-being [13]. Stockholm is one of the long-term case studies for the Stockholm Resilience Centre school of urban social-ecological research [14], and the city has four universities with an interest in people-nature interactions and several platforms for knowledge exchange and co-creation. Stockholm is rapidly growing in terms of the number of inhabitants, which is putting pressure on infrastructural development, where currently the focus is on densification. The combination of urban development and different housing types, together with the physical layout of street networks and green space qualities, influence how individual green spaces are perceived and put to use. The multiple values and different uses of publicly accessible green space in Stockholm have been cataloged and described according to a defined set of dimensions and methods, producing a sociotope map [15,16]. Drawing on a set of 30 use value concepts (e.g. enjoying wilderness, events and playgrounds) [15], the sociotopes are meant to capture the commonly experienced nature of a place by a specific group of people, the latter broadly defined as the residents of Stockholm. The sociotope map thus adds qualitative information on the different affordances of the individual green spaces [15]. To avoid the loss of desired green space qualities and minimize the related conflicts of interests between the different stakeholders, municipalities in and around Stockholm have tested participatory solutions, such as workshops with citizens and increased collaboration between planners and developers [17,18]. The results of our study could provide further inputs for such debates. The first map was created in 2003, then updated in 2009 and 2014. In this study, we used only the data from 2014 as the correlation between sociotope indications in years 2009 and 2014 was high, and because some apartment buyers could have anticipated some of the changes.

Stockholm offers suitable conditions for performing a hedonic pricing study integrated with a detailed assessment of urban green spaces due to the availability of a detailed sociotope map. It is also reasonable to assume that Stockholmers are aware of the benefits of living close to green spaces and are financially capable of paying for them. Sweden is both wealthy and advanced in environmental awareness (cf. [19]). Surprisingly, to the best of our knowledge, the city of Stockholm has been the subject of very few hedonic pricing analyses, and these have not focused on the value of urban green spaces [20,21]. Geographically, the closest hedonic pricing study focused on urban green spaces was conducted in the Jönköping region, located in the south central part of Sweden [22]. With the use of geographically weighted regression, this study reinforced prior findings that open landscape amenities positively contribute to house prices. Another related study investigated the value of green spaces in Malmö [23]. Meanwhile, several studies performed in Stockholm revealed that green spaces are in general highly valued and extensively used by the inhabitants [24–26].

### Hedonic pricing in the context of different green space characteristics

Although the valuation of green spaces has been the most popular application of hedonic pricing since the 1970s [27–29], only in the last decade has it been used to identify the specific sources of value of green spaces. Such attempts focused either on implementing more sophisticated variables which merged information on different aspects of green space provisioning (e.g. distance, accessibility, size) [30,31] or on introducing more detailed information on the benefits provided by green spaces [9,10,32,33]. Several attempts have been made to distinguish the impact of different ecosystem services [11,34,35], perceived attractiveness [9,10], biocultural value [36], the built-in infrastructure [37] and others.

Panduro and Veie [33] authored one of the first studies based on the assumption of the heterogeneity of green spaces in terms of the services they provide. Based on criteria of external, internal and social accessibility, as well as maintenance and neighboring land use, they classified green spaces and water bodies in Aalborg (Denmark) into eight groups and found that the economic impact of parks and lakes, which both have high recreational potential, is relatively higher than that of the other types of green spaces. Ham et al. [32] verified the economic effect of different land management activities on real estate prices in and around Pike National Forest (PNF) (United States). They found that an increase in the mean distance to PNF reduces the property price, but so does the adjacency (being within two miles) to noise-intensive activities (such as timber harvesting). Two hedonic pricing studies from China integrated hedonic pricing with different assessments of urban green spaces based on landscape metrics. Kong et al. [30] characterized green spaces in Jinan City based on their attractiveness, fragmentation, aggregation and the relationship between size and distance. The work of Xu et al. [31] depicted various spatial characteristics of green spaces in Beijing. Apart from distances to green spaces (indicating their availability), the authors recognized the richness of green spaces in a given geographical space, the measure of fragmentation and even the shape of green spaces.

With this article, we attempt to further contribute to the ongoing discussion on the value of the different characteristics of urban green spaces by introducing detailed information from sociotope mapping. We focus on the physical characteristics and infrastructural components of green spaces recognized by experts involved in the sociotope project, and denoted by Ståhle [12] as “use values”. We assumed that the detailed green space characteristics provided in the sociotope (e.g. “forest feeling” or “bobsleighing”) would not be recognized in terms of impacts on property prices. Following the Sociotope Handbook [12], we aggregated them thematically into five categories: “aesthetics”, “nature”, “physical activity”, “play”, and “social” (Table 1). Sociotope Handbook [12] recognized six categories: aesthetics, nature, physical activity, play, social and water. However, as our focus was on green spaces and water is usually closely connected with green, we decided to divide the category “water” into its specific attributes related to the other five categories. Note that we also included “water bodies” as yet another environmental variable, independent from the sociotope categories. For each sociotope category, we set the threshold of representativeness based on the average number of green space characteristics and the assumption that each category should be represented by a similar number of green spaces. This allowed us to determine which sociotope categories each green space represented. The thresholds for each of the five categories are listed in S1 Text, formula 2.

**Table 1:** Aggregation of characteristics recognized in sociotope.

Aggregated category	Detailed green space characteristics	Number of green spaces that best represent a given category
Aesthetics	Flower richness, Peacefulness, Vistas, Water contact	85
Nature	Forest feeling, Green oasis, Nature experience	65
Physical activity	Ballgame, Boating/boats, Jogging/running, Outdoor gym, Pool, Skating, Walks	127
Play	Ball play, Bobsleighing, Nature playground, Outdoors bath, Park playground, Playground, Skateboard/BMX, Water play	154
Social	Animal keeping/husbandry, Barbeque, Cultivation, Event, Folk life, Outdoor café, Outdoor market, Picnic area	163

In the first part of the study, we measured the effect of the green space that out of five categories is best representative for a given category. The green space was considered as representative mostly to a given category if the number of indications in this category passed the aforementioned threshold and was further from the mean than for any other category (for a more detailed explanation, see S1 Text). On this basis, we could assign each green space to one of five sociotope categories or rate it as non-representative to any category. In the second part of the study, we skipped this step and simply calculated the number of categories each green space is representative of (from zero to five), which we interpreted as the “level of multifunctionality”. This measure was the basis of categorizing green spaces in the second part of the study. The details of assigning the green spaces to categories and levels of multifunctionality can be found in S1 Text.

We focused on those green space benefits which can only be derived when users are physically present in a given place. Therefore, our spatial analysis is based on walking distances. The walking distances to the nearest green space which is representative of each sociotope category and of each “level of multifunctionality” (for the first and the second part of the study, respectively) were calculated with the Qgis 2.16.3 plugin PST [38]. Each stage of the study has a separate hedonic pricing model.

### The dataset and variables

The dataset on the prices and characteristics of 173,052 properties sold in the years 2005–2015 was acquired from Mäklarstatistik AB (Sweden), which at present is the only source available for Swedish data on real estate sales. It is worth noting that Mäklarstatistik AB is the provider of data for Statistics Sweden, a government agency which produces official statistics. According to Mäklarstatistik AB, their data cover approximately 95 percent of all real estate sales in Sweden. The dataset consists of self-reported information from individual real estate agents. The dataset includes information on individual sales of apartments and houses. For all sales, we further had information on the contract price, contract date, living area, number of rooms, monthly fee (apartments), plot size (houses), number of floors, elevator, balcony, central heating, floor number (apartments) and construction year. The information was also available on the form of tenancy: full ownership or partial ownership, e.g. within a housing cooperative. The housing development in Stockholm has followed several different planning paradigms and architectonical ideals over time, and instead of using the age of the building as a continuous variable, we classified each building as belonging to one of eleven recognized architectural epochs (listed in Table 2). The epochs differ in both the exterior and interior design of the buildings, and their approach to the design of larger neighborhoods.

**Table 2:** List of variables.

Variable	Mean	St. dev.	Exp. sign	Unit of measurement	Description
INFLATED_PRICES_M2	30334.59	11544.45	n/a	SEK	Dependent variable: transaction prices per 1 m^2^ of living area deflated by “Real estate price index for one- and two-dwelling buildings for permanent living (1981 = 100) by region and quarter” (source: http://www.statistikdatabasen.scb.se)
QUARTER_1_05 … QUARTER_4_15	n/a	n/a	n/a	n/a	Dummy variables indicating the quarter of transaction from the first quarter of 2005 to the fourth quarter of 2015
LIVING_AREA	67.50	34.57	-	m^2^	Living area of the apartment or house
SINGLE_PLOT_MINUS_LIVING	31.24	142.46	+	m^2^	The non-living area of a single house (the difference between the plot area and the living area); 0 for apartments and terraced houses
TERRACED_PLOT_MINUS_LIVING	4.92	33.87	+	m^2^	The non-living area of a terraced house (the difference between the plot area and the living area); 0 for apartments and single houses
NUMBER_OF_ROOMS	2.59	1.39	+	n/a	Number of rooms (without kitchen, bathrooms, halls)
OWNERSHIP	0.09	0.28	+	n/a	Dummy variable indicating the form of the property (1 if "ownership", 0 if other)
CONSTRUCTION_PERIOD_1500_1650 … CONSTRUCTION_PERIOD_2011_2015	n/a	n/a	n/a	n/a	Dummy variables indicating the historical period in which the real estate was built: 1500–1650; 1651–1750; 1751–1810; 1811–1900; 1901–1910; 1911–1920; 1921–1930; 1931–1970; 1971–2000; 2001–2010; 2011–2015
GEN_REN_AFTER_2010	0.0004	0.02	+	n/a	Dummy variable indicating the general renovation of real estate in 2010 and later
GEN_REN_BEFORE_2010	0.0012	0.04	+	n/a	Dummy variable indicating the general renovation of real estate before 2010
ELEVATOR	0.56	0.50	+	n/a	Dummy variables indicating the presence of structural amenities in the building (present only in the case of apartments)
BALCONY	0.07	0.25	+	n/a	
CENTRAL_HEATING	0.22	0.41	+	n/a	
FLOOR_MINUS_2 … FLOOR_24	n/a	n/a	n/a	n/a	Dummy variables indicating the floor on which the apartment is located (where ground level is treated as FLOOR_0)
KINDERGARTEN	1120.45	880.86	-	m	Walking distances to educational facilities
SCHOOL	1140.16	784.27	-	m	
UNIVERSITY	4561.05	2771.55	-	m	
CINEMA	2543.16	1845.03	-	m	Walking distances to cultural and social facilities
THEATRE	2324.68	2303.06	-	m	
ARTS_CENTER	4957.60	3538.70	-	m	
COMMUNITY_CENTER	4071.05	1860.53	-	m	
SWIMMING_POOL	5954.88	2866.40	-	m	Walking distance to the nearest swimming pool
PENDELSTATION	2679.75	1633.53	-	m	Walking distance to the nearest ‘pendelstation’ or subway station (pendel-trains are suburban commuter trains and reach farther than the subway).
SUBWAY	979.40	718.73	-	m	
CENTRAL_STATION	4742.66	3403.90	-	m	Walking distance to Stockholm Central Station, marking both the transport hub and a conjectural central point of the city
SOCIOTOPE_AESTHETICS	1548.57	1369.16	-	m	Walking distance to the border of the nearest green space representing a given sociotope category (used in the first stage of the study)
SOCIOTOPE_NATURE	1856.56	1108.96	-	m	
SOCIOTOPE_PHYSICAL	610.21	520.07	-	m	
SOCIOTOPE_PLAY	701.69	496.64	-	m	
SOCIOTOPE_SOCIAL	688.89	762.18	-	m	
MULTIFUNCTIONAL_0	198.84	168.07	-	m	Walking distance to the border of the nearest green space representing a given level of multifunctionality (used in the second stage of the study)
MULTIFUNCTIONAL_1	323.78	255.97	-	m	
MULTIFUNCTIONAL_2	685.53	507.65	-	m	
MULTIFUNCTIONAL_3	1297.40	928.98	-	m	
MULTIFUNCTIONAL_4	2085.90	1886.39	-	m	
MULTIFUNCTIONAL_5	5547.13	3862.62	-	m	
WATER	1154.92	807.99	-	m	Walking distance to the nearest water body
GREENERY_BUF_500	21.91	11.19	+	%	The share of greenery in the buffer of 500 m

The dataset also includes geographical coordinates and address information. Since the main purpose of our study involves a geographically-based analysis, it is essential that this information be as accurate as possible. Due to the fact that the coordinate information from Mäklarstatistik is based on a “drag-and-drop” system, in which each individual real estate agent must pinpoint the location on a map, there is considerable room for error. We thus decided to use the address information of the sales instead. In order to do so, we made use of Google’s geocoding service to transform the addresses into geographical coordinates. Hence, we have two points of geographical reference for the sale location, which allows us to improve the geographical precision. Based on this coordinate information we excluded the observations for which the points of reference differed by more than 100 meters.

Drawing from this dataset, we constructed a set of variables which can be classified into three groups: structural, locational, environmental. The structural variables are the basic characteristics of the apartments and houses, such as living area or the age of the building. Many of these variables were introduced in the form of dummy variables (e.g. the age of the building, the floor) as we could not assume a linear relationship between these characteristics and the real estate prices. The set of locational variables consists of walking distances (measured in meters) to educational, cultural, social and sports facilities, as well as transport hubs. Walking distances to green spaces as well as the share of greenery in the 500 m buffer around the property represent the set of environmental variables. The reason for choosing the 500 m buffer was that the other buffers tested gave a worse fitness of the model (measured by the residual variance). Table 2 presents the full list of variables. The dummy variables indicating the quarter in which the transaction took place, the construction period, and the floor are accompanied by a description in Table 2, but to save space the descriptive statistics for all of these variables are omitted.

### Econometric analysis

A hedonic pricing study involves the estimation of the parameters of the following model:

P=Sα+Lβ+Eγ+ε

where **P** is the vector of real estate prices, **S**, **E** and **L** are matrices of, respectively, structural, locational and environmental variables, **α**, **β**, and **γ** are vectors of parameters for those variables and **ε** is the random error vector. The use of spatial data might cause spatial autocorrelation [39]; therefore, we performed the Lagrange Multiplier tests which revealed that we were dealing with both an autocorrelated explained variable and an autocorrelated error term. We dealt with those problems with the use of the spatial autoregressive model with autoregressive disturbance (SARAR) [40]. It means we had to enlarge our primary model to the following form:

P=ρWP+Sα+Lβ+Eγ+ε

ε=λWε+μ

$$\mu ∼N(0;\sigma^{2}I)$$

where **W** is the spatial weights matrix, **ε** becomes the vector of spatially autoregressive errors, ***μ*** is the vector of random errors, ρ is the coefficient measuring the autoregression of the dependent variable and λ is the coefficient measuring the autoregression of the error **ε**. Of the 5-, 10-, and 15- nearest neighbors spatial weights matrices, the last one minimized the residual variance. In models with the spatially lagged dependent variable, it is possible to recognize the direct, indirect and total effects [41,42]. In our case, the direct effect is the change of the property price caused solely by the change of the attributes of the property itself. The indirect effect represents the change of the property price caused by the changes in the prices of the neighboring properties (defined by the spatial weights matrix–in our case, the five nearest neighbors) which are caused by the changes of their attributes. The direct and indirect effects sum up to the total effect. In the discussion, we will analyze only the total effects. The thorough analysis of the model diagnostics indicated the model that fits our data generating process best (based on residual variance) is the log-log model. It means that both the dependent variable and some of the independent variables were logarithmized which, apart from improving the model accuracy, allows parameters to be interpreted as elasticities. The parameters were estimated with the generalized method of moments [43] in R x64 3.3.1.

## Results

### Stage I: The monetary value of different green space characteristics

Table 3 shows selected results from the first part of the study (the list of all estimates, including the results for each quarter of transactions and each floor, can be found in S1 Table). The impacts of the structural variables conform with general expectations. The effect of increasing the distance to educational and cultural objects is less intuitive: kindergartens are treated as disamenities, schools turn out to be insignificant while universities increase property prices. The positive effect of universities might be caused by the feeling of elitism associated with those buildings. The same explanation might apply to the positive effect of theatres and arts centers. The model revealed that green spaces representative of the categories “aesthetics”, “social” and “nature” are perceived as amenities by real estate buyers. The green spaces qualified as being representative of the category “physical activity” are insignificant in explaining the property prices while those assigned to category “play” seem to be disamenities. Water bodies also have a positive and strong impact on property prices, but not as strong as green spaces assigned to the “aesthetics” sociotope category. Surprisingly, an additional percentage point of the share of greenery in the 500-meter buffer is associated with a decrease in property prices.

**Table 3:** Results of the first stage of the study (focused on the representativeness of green spaces to sociotope categories).

	Direct	Sig.	Indirect	Sig.	Total	Sig.
**Results for quarters**	**(in S1 Table)**		**(in S1 Table)**		**(in S1 Table)**	
LIVING_AREA	-0.0031	***	-0.0026	***	-0.0057	***
SINGLE_PLOT_MINUS_LIVING	0.0002	***	0.0002	***	0.0004	***
TERRACED_PLOT_MINUS_LIVING	0.0001	***	0.0001	***	0.0002	***
NUMBER_OF_ROOMS	0.0225	***	0.0191	***	0.0416	***
OWNERSHIP	0.2717	***	0.2311	***	0.5028	***
CONSTRUCTION_PERIOD_1500_1650	0.0813	***	0.0691	***	0.1504	***
CONSTRUCTION_PERIOD_1651_1750	0.1073	***	0.0913	***	0.1986	***
CONSTRUCTION_PERIOD_1751_1810	-0.0087		-0.0074		-0.0162	
CONSTRUCTION_PERIOD_1811_1900	-0.0047		-0.0040		-0.0087	
CONSTRUCTION_PERIOD_1901_1910	-0.0064		-0.0055		-0.0119	
CONSTRUCTION_PERIOD_1911_1920	0.0083		0.0070		0.0153	
CONSTRUCTION_PERIOD_1921_1930	-0.0316	***	-0.0269	***	-0.0584	***
CONSTRUCTION_PERIOD_1931_1970	-0.0547	***	-0.0465	***	-0.1011	***
CONSTRUCTION_PERIOD_1971_2000	-0.1510	***	-0.1284	***	-0.2794	***
CONSTRUCTION_PERIOD_2001_2010	-0.0066		-0.0057		-0.0123	
GEN_REN_AFTER_2010	0.1404	***	0.1194	***	0.2598	***
GEN_REN_BEFORE_2010	-0.0172		-0.0146		-0.0318	
ELEVATOR	0.0083	***	0.0071	***	0.0154	***
BALCONY	-0.0027	*	-0.0023	*	-0.0050	*
CENTRAL_HEATING	0.0001		0.0001		0.0002	
**Results for floors**	**(in S1 Table)**		**(in S1 Table)**		**(in S1 Table)**	
ln(KINDERGARTEN)	0.0028	*	0.0024	*	0.0051	*
ln(SCHOOL)	-0.0004		-0.0004		-0.0008	
ln(UNIVERSITY)	-0.0323	***	-0.0275	***	-0.0598	***
ln(CINEMA)	-0.0345	***	-0.0294	***	-0.0639	***
ln(THEATRE)	-0.0514	***	-0.0437	***	-0.0951	***
ln(ARTS_CENTER)	-0.0826	***	-0.0702	***	-0.1528	***
ln(COMMUNITY_CENTER)	0.0303	***	0.0257	***	0.0560	***
ln(SWIMMING_POOL)	0.0344	***	0.0292	***	0.0636	***
ln(PENDELSTATION)	-0.0010		-0.0009		-0.0019	
ln(SUBWAY)	-0.0055	***	-0.0046	***	-0.0101	***
ln(CENTRAL_STATION)	0.0001		0.0000		0.0001	
ln(SOCIOTOPE_AESTHETICS)	-0.0320	***	-0.0272	***	-0.0593	***
ln(SOCIOTOPE_NATURE)	-0.0052	***	-0.0044	***	-0.0096	***
ln(SOCIOTOPE_PHYSICAL)	0.0010		0.0009		0.0019	
ln(SOCIOTOPE_PLAY)	0.0121	***	0.0103	***	0.0224	***
ln(SOCIOTOPE_SOCIAL)	-0.0101	***	-0.0086	***	-0.0187	***
ln(WATER)	-0.0186	***	-0.0158	***	-0.0343	***
GREENERY_BUF_500	-0.0003	***	-0.0003	***	-0.0006	***
Rho	0.469	***				
Lambda	0.615	n/a				
Residual variance (sigma squared)	0.024	n/a				
Number of observations	173052	n/a				

***—significant at 10% level

**—significant at 5% level

*—significant at 1% level

### Stage II: The monetary value of multifunctionality

**Fig 1:**
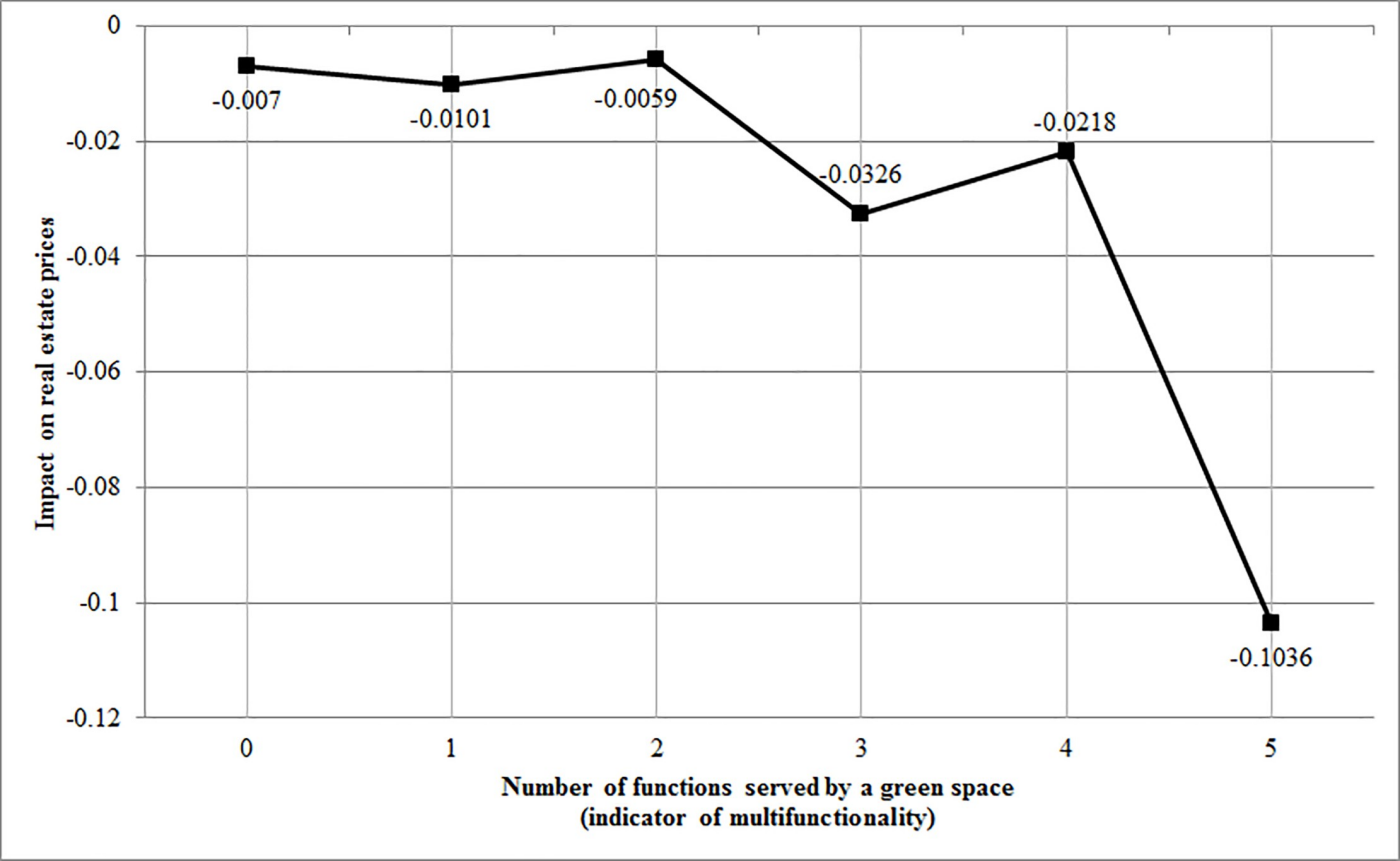
Fall in the price of real estate in reaction to an increase in the distance to a green space characterized by a given level of multifunctionality.

Table 4 lists selected results from the second stage of the study (all results are listed in S2 Table). The impacts of structural and locational variables seem consistent with the results of the first model. The results for the green spaces in the second part of the study have shown the directed yet fluctuated gradation of the impact of green spaces on real estate prices, with the most multifunctional being the most desired by real estate buyers. A 1% increase in the distance to the nearest green space without any sociotope characteristics decreases the property price by only 0.7% whereas the same 1% increase in the distance to the nearest green space with all five characteristics decreases the property price by as much as 10.4%. The increase of the economic effect with every additional function is depicted in Fig 1.

**Table 4:** Results of the second stage of the study (focused on the multifunctionality of green spaces).

	Direct	Sig.	Indirect	Sig.	Total	Sig.
**Results for quarters**	**(in S2 Table)**		**(in S2 Table)**		**(in S2 Table)**	
LIVING_AREA	-0.0032	***	-0.0010	***	-0.0042	***
SINGLE_PLOT_MINUS_LIVING	0.0002	***	0.0001	***	0.0003	***
TERRACED_PLOT_MINUS_LIVING	0.0001	***	0.00003	***	0.0001	***
NUMBER_OF_ROOMS	0.0228	***	0.0073	***	0.0302	***
OWNERSHIP	0.3136	***	0.1005	***	0.4141	***
CONSTRUCTION_PERIOD_1500_1650	0.0784	***	0.0251	***	0.1035	***
CONSTRUCTION_PERIOD_1651_1750	0.0816	***	0.0261	***	0.1077	***
CONSTRUCTION_PERIOD_1751_1810	-0.0021		-0.0007		-0.0028	
CONSTRUCTION_PERIOD_1811_1900	-0.0160	***	-0.0051	***	-0.0211	***
CONSTRUCTION_PERIOD_1901_1910	-0.0236	***	-0.0076	***	-0.0312	***
CONSTRUCTION_PERIOD_1911_1920	-0.0133	**	-0.0043	**	-0.0176	**
CONSTRUCTION_PERIOD_1921_1930	-0.0491	***	-0.0157	***	-0.0648	***
CONSTRUCTION_PERIOD_1931_1970	-0.0857	***	-0.0275	***	-0.1132	***
CONSTRUCTION_PERIOD_1971_2000	-0.1917	***	-0.0614	***	-0.2532	***
CONSTRUCTION_PERIOD_2001_2010	-0.0207	***	-0.0066	***	-0.0274	***
GEN_REN_AFTER_2010	0.1734	***	0.0556	***	0.2290	***
GEN_REN_BEFORE_2010	-0.0086		-0.0027		-0.0113	
ELEVATOR	0.0093	***	0.0030	***	0.0123	***
BALCONY	-0.0047	***	-0.0015	***	-0.0061	***
CENTRAL_HEATING	-0.0007		-0.0002		-0.0010	
**Results for floors**	**(in S2 Table)**		**(in S2 Table)**		**(in S2 Table)**	
ln(KINDERGARTEN)	-0.0015		-0.0005		-0.0019	
ln(SCHOOL)	0.0007		0.0002		0.0010	
ln(UNIVERSITY)	-0.0167	***	-0.0053	***	-0.0220	***
ln(CINEMA)	-0.0439	***	-0.0141	***	-0.0580	***
ln(THEATRE)	-0.0557	***	-0.0179	***	-0.0736	***
ln(ARTS_CENTER)	-0.1289	***	-0.0413	***	-0.1702	***
ln(COMMUNITY_CENTER)	-0.0019		-0.0006		-0.0025	
ln(SWIMMING_POOL)	0.0637	***	0.0204	***	0.0841	***
ln(PENDELSTATION)	0.0077	***	0.0025	***	0.0101	***
ln(SUBWAY)	-0.0004		-0.0001		-0.0005	
ln(CENTRAL_STATION)	-0.0002		0.0000		-0.0002	
ln(MULTIFUNCTIONAL_0)	-0.0053	***	-0.0017	***	-0.0070	***
ln(MULTIFUNCTIONAL_1)	-0.0077	***	-0.0025	***	-0.0101	***
ln(MULTIFUNCTIONAL_2)	-0.0045	***	-0.0014	***	-0.0059	***
ln(MULTIFUNCTIONAL_3)	-0.0247	***	-0.0079	***	-0.0326	***
ln(MULTIFUNCTIONAL_4)	-0.0165	***	-0.0053	***	-0.0218	***
ln(MULTIFUNCTIONAL_5)	-0.0784	***	-0.0251	***	-0.1036	***
ln(WATER)	-0.0251	***	-0.0080	***	-0.0331	***
GREENERY_BUF_500	-0.0019	***	-0.0006	***	-0.0026	***
Rho	0.2503	***				
Lambda	0.5772	n/a				
Residual variance (sigma squared)	0.0261	n/a				
Number of observations	173052	n/a				

***—significant at 10% level

**—significant at 5% level

*—significant at 1% level

## Discussion and conclusions

Although hedonic pricing is a popular method for valuing urban green spaces, and although in most cases hedonic pricing studies have confirmed positive impacts of green spaces on real estate prices, only recently has hedonic pricing started to be used to study the value of specific characteristics of urban green spaces. Some categories of green spaces have been found to have negative impacts on real estate prices, in particular, cemeteries [11,44,45], and based on a number of detailed studies, some general guesses could be made regarding which green space features attracted real estate buyers. Our study provides a more detailed indication of what matters to real estate buyers, although hedonic pricing studies always have to be considered in the relevant local context.

The results from the first stage of the study did not support the first part of our hypothesis for the first stage of the study: not all characteristics of green spaces seem desired by real estate buyers in Stockholm. However, the effect of various green space categories differs, which is in line with our hypothesis. The most desired category was “aesthetics”. Green spaces assigned to the “social” category had a slightly weaker effect on property prices. Green spaces aligning most strongly with the “nature” attribute exerted the weakest, yet still positive, effect on property prices. The “play” category had a negative impact on property prices while “physical activity” was statistically insignificant.

Even though this study is, to our knowledge, the first to directly address the aspect of aesthetics of urban green spaces with the use of hedonic pricing, we can assume that this characteristic was important for real estate buyers in Jinan City in China, where scenery forests turned out to be the most desired green spaces by real estate buyers [30]. However, our results are, in general, somehow contrary to the results obtained by Brander and Koetse [46], who in their meta-analysis of contingent valuation found that among aesthetics, preservation and recreational opportunities, only the latter significantly increase the economic value of a green space.

The “social” attribute has not, as far as we are aware, been the subject of hedonic pricing research. However, our result does not seem surprising in the light of results obtained by Cohen et al. [47]. Their analysis of park use in Southern California showed that the park area and the number of activities organized there were the most significant characteristics that increased the number of park users.

The significance of the “nature” attribute is not without precedent either. In their hedonic pricing study, Lutzenhiser and Netusil [48] found that among golf courses, cemeteries, urban parks, specialty parks and natural area parks, the latter had the largest statistically significant effect on property prices in Portland. Similarly, Tyrvainen and Miettinen ([35]: p. 211) found that a forested area characterized as “important for screening and pollution control” had a significant positive impact on property prices while a wooded recreational area providing mainly recreational opportunities did not. The study conducted in Stockholm by Samuelsson et al. [49] showed that areas with high natural temperature regulating capacities have very high rates of positive experiences. However positive and significant, the nature characteristic had the weakest role in increasing property prices, which indicates that–especially in some local contexts–“green” seems to be an auxiliary aspect of urban green spaces in terms of the role they play in urban life and the value they add. This supports the social-ecological vision of urban green spaces.

The possible explanation for the negative impact of the “play” attribute might be that green spaces characterized mainly by this attribute happened to be the smallest and the most numerous. The abundance of these green spaces, in combination with their sizes, might deprive them of any sort of worth-paying-for exclusiveness. Finally, the lack of significance of the “physical activity” category might be attributed to the fact that the city of Stockholm provides opportunities for such activities, not only in green spaces but also along the numerous waterfront walkways. Therefore, those green spaces might not constitute any extra value for real estate buyers.

In order to evaluate the robustness of the results, we performed sensitivity analysis in which we moved the most non-obvious characteristics to different categories (“ballgame” and “skating” were assigned to category “play” instead of “physical activity”, and “skateboard” was assigned to “physical activity” instead of “play”). The results from these three models showed the robustness of the results for all the categories (in terms of the sign and statistical significance) except for “physical activity”, which became an amenity in the first two cases. In the third case, the results were fully consistent with the baseline model.

The decrease of property price due to growing share of greenery in the 500 m radius might be caused by the higher abundance of large green spaces in the suburbs than in the expensive downtown. However, this relation might be specific to Stockholm, a city well-endowed with green spaces that, additionally, are relatively evenly distributed (with the size of green spaces growing towards the boundaries of the city).

We see the opportunity to further elaborate on the valuation of the functions of urban green spaces by analyzing the impact of specific combinations of their characteristics. This analysis might even better reflect the way real estate buyers conceptualize green spaces, compared with the “one function per green space” approach we employed in the first part of our study.

The second part of the study provided support for the hypothesis that the more multifunctional the green space is, the greater the economic impact it has on real estate prices. This result is broadly in line with the results of Kimpton [50], who performed a spatial analysis of green spaces in Brisbane (Australia), focusing on their size and shape but also on the provided amenities. Kimpton collected 73 keywords describing green spaces (e.g. “barbecue”, “toilet”, “dog”, “playground”) and grouped them into ten general categories. He then examined whether the inequities in the provision of amenities could be explained by the social composition of the neighborhood and drew conclusions from the discovered correlates. The results showed that “affluent neighborhoods have an abundance of high amenity greenspace” [p. 137]. The economic value of multifunctionality adds to the long list of its benefits recognized by Connop et al. [4], which includes a contribution to urban resilience and responsiveness to challenges such as overheating, flooding or air pollution. Our study fits well in the framework created by Hansen and Pauleit [51], who indicated the recognition of stakeholders’ preferences towards multifunctionality as an important step in building long-term spatial planning strategies. The results we obtained support this conclusion and could be used as a guidance for local policies.

The comparison between the two stages of the study brings interesting observations: while all “levels of multifunctionality” were associated with a positive impact on property prices, the same could not be said about all “sociotope categories”. It is important to remember that the same green spaces were analyzed in both stages of the study, only they were classified in different ways. This shows that applying detailed categorization can bring unique information on preferences regarding urban green space characteristics. The possibility to use such detailed information as a basis for hedonic pricing indeed makes this specific application of the hedonic pricing method a relatively close parallel to the typically more flexible choice experiment, yet still referring to revealed rather than stated preferences only.

## Supporting information

S1 Text Assigning green spaces to categories and levels of multifunctionality. (DOCX)

S1 Table Results of the first stage of the study (focused on the representativeness of green spaces to the sociotope categories). (DOCX)

S2 Table Results of the second stage of the study (focused on the multifunctionality of green spaces). (DOCX)
