# Supplementary material for: Valuing individual characteristics and the multifunctionality of urban green spaces: The integration of sociotope mapping and hedonic pricing
Source: PLoS One. 2019 Mar 6;14(3):e0212277. doi: 10.1371/journal.pone.0212277 (PMC6402650; doi:10.1371/journal.pone.0212277)
Supplement: S1 Text — (DOCX) [file pone.0212277.s001.docx]

# S1 Text. **Assigning green spaces to categories and levels of multifunctionality**

$X_{\left( s,g \right)}$ – number of characteristics belonging to category *s*, where $s\in\{„\text{aesthetics”; „nature”; „physical activity”; „play”; „social”}\text{\}}$ in green space *g*, where $g\in<1;\ldots;1377>$

max($X_{\left( s,g \right)})$ = $\left\{ \begin{aligned} 4 \mathrm{for} s="aesthetics" \\ 3 \mathrm{for} s="\text{nature}\text{"} \\ 7 \mathrm{for} s="\text{physical}\text{ }\text{activity}\text{"} \\ 8 \mathrm{for} s="\text{play}\text{"} \\ 8 \mathrm{for} s="social" \end{aligned} \right.$ (1)

The green space *m* is *representative* to category *a* when

$X_{\left( a,m \right)}$ is higher than $\left\{ \begin{aligned} 2 \mathrm{for} a="aesthetics" \\ 1 \mathrm{for} a="\text{nature}\text{"} \\ 1 \mathrm{for} a="\text{physical}\text{ }\text{activity}\text{"} \\ 2 \mathrm{for} a="\text{play}\text{"} \\ 1 \mathrm{for} a="social" \end{aligned} \right.$ (2)

These thresholds were determined on the basis of the average number of characteristics in a given category (1) and the assumption that each category should be represented by a similar number of green spaces.
The green space *m* is *mostly representative* to the category *a* when it is *representative* (2) to the category *a* and

$$\frac{X_{\left( a,m \right)}}{\frac{\sum_{i=1}^{1377} X_{\left( a,i \right)}}{1377}}>\frac{X_{\left( c,m \right)}}{\frac{\sum_{i=1}^{1377} X_{\left( c,i \right)}}{1377}}$$

for every $c\neq a$ (3)

The multifunctionality of green space analyzed in the second stage of the study is equal to *d* when the green space is *representative* to *d* categories according to (2).
