# Supplementary material for: Valuing individual characteristics and the multifunctionality of urban green spaces: The integration of sociotope mapping and hedonic pricing
Source: PLoS One. 2019 Mar 6;14(3):e0212277. doi: 10.1371/journal.pone.0212277 (PMC6402650; doi:10.1371/journal.pone.0212277)
Supplement: S1 Table — (DOCX) [file pone.0212277.s002.docx]

# S1 Table. **Results of the first stage of the study (focused on the representativeness of green spaces to the sociotope categories)**

|  | Direct | Sig. | Indirect | Sig. | Total | Sig. |
| --- | --- | --- | --- | --- | --- | --- |
| QUARTER_2_05 | 0.0117 | ** | 0.0099 | ** | 0.0216 | ** |
| QUARTER_3_05 | 0.0472 | *** | 0.0401 | *** | 0.0873 | *** |
| QUARTER_4_05 | 0.0491 | *** | 0.0417 | *** | 0.0908 | *** |
| QUARTER_1_06 | 0.0753 | *** | 0.0641 | *** | 0.1394 | *** |
| QUARTER_2_06 | 0.0661 | *** | 0.0562 | *** | 0.1223 | *** |
| QUARTER_3_06 | 0.0731 | *** | 0.0622 | *** | 0.1353 | *** |
| QUARTER_4_06 | 0.0591 | *** | 0.0503 | *** | 0.1094 | *** |
| QUARTER_1_07 | 0.1406 | *** | 0.1196 | *** | 0.2602 | *** |
| QUARTER_2_07 | 0.1307 | *** | 0.1111 | *** | 0.2418 | *** |
| QUARTER_3_07 | 0.1153 | *** | 0.0981 | *** | 0.2134 | *** |
| QUARTER_4_07 | 0.0314 | *** | 0.0267 | *** | 0.0582 | *** |
| QUARTER_1_08 | 0.0738 | *** | 0.0628 | *** | 0.1367 | *** |
| QUARTER_2_08 | 0.0841 | *** | 0.0716 | *** | 0.1557 | *** |
| QUARTER_3_08 | 0.0163 | *** | 0.0138 | *** | 0.0301 | *** |
| QUARTER_4_08 | -0.0610 | *** | -0.0518 | *** | -0.1128 | *** |
| QUARTER_1_09 | 0.0606 | *** | 0.0516 | *** | 0.1122 | *** |
| QUARTER_2_09 | 0.0403 | *** | 0.0343 | *** | 0.0747 | *** |
| QUARTER_3_09 | 0.0554 | *** | 0.0471 | *** | 0.1024 | *** |
| QUARTER_4_09 | 0.0652 | *** | 0.0555 | *** | 0.1207 | *** |
| QUARTER_1_10 | 0.0574 | *** | 0.0488 | *** | 0.1061 | *** |
| QUARTER_2_10 | 0.0116 | *** | 0.0099 | *** | 0.0215 | *** |
| QUARTER_3_10 | 0.0479 | *** | 0.0408 | *** | 0.0887 | *** |
| QUARTER_4_10 | 0.0762 | *** | 0.0648 | *** | 0.1411 | *** |
| QUARTER_1_11 | 0.0954 | *** | 0.0812 | *** | 0.1766 | *** |
| QUARTER_2_11 | 0.0589 | *** | 0.0501 | *** | 0.1089 | *** |
| QUARTER_3_11 | 0.0479 | *** | 0.0407 | *** | 0.0887 | *** |
| QUARTER_4_11 | 0.0741 | *** | 0.0630 | *** | 0.1372 | *** |
| QUARTER_1_12 | 0.1050 | *** | 0.0893 | *** | 0.1944 | *** |
| QUARTER_2_12 | 0.1089 | *** | 0.0926 | *** | 0.2015 | *** |
| QUARTER_3_12 | 0.1117 | *** | 0.0950 | *** | 0.2067 | *** |
| QUARTER_4_12 | 0.1232 | *** | 0.1048 | *** | 0.2280 | *** |
| QUARTER_1_13 | 0.1542 | *** | 0.1312 | *** | 0.2854 | *** |
| QUARTER_2_13 | 0.1576 | *** | 0.1341 | *** | 0.2917 | *** |
| QUARTER_3_13 | 0.1803 | *** | 0.1534 | *** | 0.3337 | *** |
| QUARTER_4_13 | 0.1813 | *** | 0.1542 | *** | 0.3355 | *** |
| QUARTER_1_14 | 0.1845 | *** | 0.1569 | *** | 0.3413 | *** |
| QUARTER_2_14 | 0.1811 | *** | 0.1541 | *** | 0.3352 | *** |
| QUARTER_3_14 | 0.1957 | *** | 0.1665 | *** | 0.3622 | *** |
| QUARTER_4_14 | 0.1888 | *** | 0.1606 | *** | 0.3494 | *** |
| QUARTER_1_15 | 0.2441 | *** | 0.2076 | *** | 0.4516 | *** |
| QUARTER_2_15 | 0.2238 | *** | 0.1904 | *** | 0.4142 | *** |
| QUARTER_3_15 | 0.2426 | *** | 0.2064 | *** | 0.4490 | *** |
| QUARTER_4_15 | 0.2199 | *** | 0.1870 | *** | 0.4069 | *** |
| LIVING_AREA | -0.0031 | *** | -0.0026 | *** | -0.0057 | *** |
| SINGLE_PLOT_MINUS_LIVING | 0.0002 | *** | 0.0002 | *** | 0.0004 | *** |
| TERRACED_PLOT_MINUS_LIVING | 0.0001 | *** | 0.0001 | *** | 0.0002 | *** |
| NUMBER_OF_ROOMS | 0.0225 | *** | 0.0191 | *** | 0.0416 | *** |
| OWNERSHIP | 0.2717 | *** | 0.2311 | *** | 0.5028 | *** |
| CONSTRUCTION_PERIOD_1500_1650 | 0.0813 | *** | 0.0691 | *** | 0.1504 | *** |
| CONSTRUCTION_PERIOD_1651_1750 | 0.1073 | *** | 0.0913 | *** | 0.1986 | *** |
| CONSTRUCTION_PERIOD_1751_1810 | -0.0087 |  | -0.0074 |  | -0.0162 |  |
| CONSTRUCTION_PERIOD_1811_1900 | -0.0047 |  | -0.0040 |  | -0.0087 |  |
| CONSTRUCTION_PERIOD_1901_1910 | -0.0064 |  | -0.0055 |  | -0.0119 |  |
| CONSTRUCTION_PERIOD_1911_1920 | 0.0083 |  | 0.0070 |  | 0.0153 |  |
| CONSTRUCTION_PERIOD_1921_1930 | -0.0316 | *** | -0.0269 | *** | -0.0584 | *** |
| CONSTRUCTION_PERIOD_1931_1970 | -0.0547 | *** | -0.0465 | *** | -0.1011 | *** |
| CONSTRUCTION_PERIOD_1971_2000 | -0.1510 | *** | -0.1284 | *** | -0.2794 | *** |
| CONSTRUCTION_PERIOD_2001_2010 | -0.0066 |  | -0.0057 |  | -0.0123 |  |
| GEN_REN_AFTER_2010 | 0.1404 | *** | 0.1194 | *** | 0.2598 | *** |
| GEN_REN_BEFORE_2010 | -0.0172 |  | -0.0146 |  | -0.0318 |  |
| ELEVATOR | 0.0083 | *** | 0.0071 | *** | 0.0154 | *** |
| BALCONY | -0.0027 | * | -0.0023 | * | -0.0050 | * |
| CENTRAL_HEATING | 0.0001 |  | 0.0001 |  | 0.0002 |  |
| FLOOR_MINUS_2 | -0.0479 | * | -0.0407 | * | -0.0886 | * |
| FLOOR_MINUS_1 | 0.0321 | * | 0.0273 | * | 0.0594 | * |
| FLOOR_1 | -0.0174 | *** | -0.0148 | *** | -0.0321 | *** |
| FLOOR_2 | 0.0065 | *** | 0.0055 | *** | 0.0120 | *** |
| FLOOR_3 | 0.0203 | *** | 0.0173 | *** | 0.0375 | *** |
| FLOOR_4 | 0.0403 | *** | 0.0343 | *** | 0.0746 | *** |
| FLOOR_5 | 0.0762 | *** | 0.0648 | *** | 0.1410 | *** |
| FLOOR_6 | 0.1102 | *** | 0.0937 | *** | 0.2039 | *** |
| FLOOR_7 | 0.1059 | *** | 0.0901 | *** | 0.1960 | *** |
| FLOOR_8 | 0.1034 | *** | 0.0879 | *** | 0.1913 | *** |
| FLOOR_9 | 0.0806 | *** | 0.0686 | *** | 0.1492 | *** |
| FLOOR_10 | 0.0983 | *** | 0.0836 | *** | 0.1818 | *** |
| FLOOR_11 | 0.1157 | *** | 0.0984 | *** | 0.2141 | *** |
| FLOOR_12 | 0.1048 | *** | 0.0892 | *** | 0.1940 | *** |
| FLOOR_13 | 0.1163 | *** | 0.0989 | *** | 0.2152 | *** |
| FLOOR_14 | 0.1413 | *** | 0.1201 | *** | 0.2614 | *** |
| FLOOR_15 | 0.1790 | *** | 0.1523 | *** | 0.3313 | *** |
| FLOOR_16 | 0.1531 | *** | 0.1302 | *** | 0.2833 | *** |
| FLOOR_17 | 0.2734 | *** | 0.2325 | *** | 0.5059 | *** |
| FLOOR_18 | 0.2701 | *** | 0.2297 | *** | 0.4998 | *** |
| FLOOR_20 | 0.1936 | ** | 0.1646 | ** | 0.3582 | ** |
| FLOOR_21 | 0.3071 | *** | 0.2612 | *** | 0.5683 | *** |
| FLOOR_22 | 0.3929 | *** | 0.3341 | *** | 0.7270 | *** |
| FLOOR_23 | 0.5186 | *** | 0.4411 | *** | 0.9596 | *** |
| FLOOR_24 | 0.3497 | ** | 0.2974 | ** | 0.6471 | ** |
| ln(KINDERGARTEN) | 0.0028 | * | 0.0024 | * | 0.0051 | * |
| ln(SCHOOL) | -0.0004 |  | -0.0004 |  | -0.0008 |  |
| ln(UNIVERSITY) | -0.0323 | *** | -0.0275 | *** | -0.0598 | *** |
| ln(CINEMA) | -0.0345 | *** | -0.0294 | *** | -0.0639 | *** |
| ln(THEATRE) | -0.0514 | *** | -0.0437 | *** | -0.0951 | *** |
| ln(ARTS_CENTER) | -0.0826 | *** | -0.0702 | *** | -0.1528 | *** |
| ln(COMMUNITY_CENTER) | 0.0303 | *** | 0.0257 | *** | 0.0560 | *** |
| ln(SWIMMING_POOL) | 0.0344 | *** | 0.0292 | *** | 0.0636 | *** |
| ln(PENDELSTATION) | -0.0010 |  | -0.0009 |  | -0.0019 |  |
| ln(SUBWAY) | -0.0055 | *** | -0.0046 | *** | -0.0101 | *** |
| ln(CENTRAL_STATION) | 0.0001 |  | 0.0000 |  | 0.0001 |  |
| ln(SOCIOTOPE_AESTHETICS) | -0.0320 | *** | -0.0272 | *** | -0.0593 | *** |
| ln(SOCIOTOPE_NATURE) | -0.0052 | *** | -0.0044 | *** | -0.0096 | *** |
| ln(SOCIOTOPE_PHYSICAL) | 0.0010 |  | 0.0009 |  | 0.0019 |  |
| ln(SOCIOTOPE_PLAY) | 0.0121 | *** | 0.0103 | *** | 0.0224 | *** |
| ln(SOCIOTOPE_SOCIAL) | -0.0101 | *** | -0.0086 | *** | -0.0187 | *** |
| ln(WATER) | -0.0186 | *** | -0.0158 | *** | -0.0343 | *** |
| GREENERY_BUF_500 | -0.0003 | *** | -0.0003 | *** | -0.0006 | *** |
|  |  |  |  |  |  |  |
| Rho | 0.469 | *** |  |  |  |  |
| Lambda | 0.615 | n/a |  |  |  |  |
| Residual variance (sigma squared) | 0.024 | n/a |  |  |  |  |
| Number of observations | 173052 | n/a |  |  |  |  |

*** - significant at 10% level, ** - significant at 5% level, * - significant at 1% level
