# Supplementary material for: Valuing individual characteristics and the multifunctionality of urban green spaces: The integration of sociotope mapping and hedonic pricing
Source: PLoS One. 2019 Mar 6;14(3):e0212277. doi: 10.1371/journal.pone.0212277 (PMC6402650; doi:10.1371/journal.pone.0212277)
Supplement: S2 Table — (DOCX) [file pone.0212277.s003.docx]

# S2 Table. **Results of the second stage of the study (focused on the multifunctionality of green spaces)**

|  | Direct | Sig. | Indirect | Sig. | Total | Sig. |
| --- | --- | --- | --- | --- | --- | --- |
| QUARTER_2_05 | 0.0123 | ** | 0.0040 | ** | 0.0163 | ** |
| QUARTER_3_05 | 0.0478 | *** | 0.0153 | *** | 0.0631 | *** |
| QUARTER_4_05 | 0.0496 | *** | 0.0159 | *** | 0.0655 | *** |
| QUARTER_1_06 | 0.0744 | *** | 0.0239 | *** | 0.0983 | *** |
| QUARTER_2_06 | 0.0656 | *** | 0.0210 | *** | 0.0867 | *** |
| QUARTER_3_06 | 0.0734 | *** | 0.0235 | *** | 0.0969 | *** |
| QUARTER_4_06 | 0.0580 | *** | 0.0186 | *** | 0.0766 | *** |
| QUARTER_1_07 | 0.1395 | *** | 0.0447 | *** | 0.1843 | *** |
| QUARTER_2_07 | 0.1288 | *** | 0.0413 | *** | 0.1701 | *** |
| QUARTER_3_07 | 0.1147 | *** | 0.0368 | *** | 0.1515 | *** |
| QUARTER_4_07 | 0.0312 | *** | 0.0100 | *** | 0.0412 | *** |
| QUARTER_1_08 | 0.0720 | *** | 0.0231 | *** | 0.0950 | *** |
| QUARTER_2_08 | 0.0828 | *** | 0.0265 | *** | 0.1093 | *** |
| QUARTER_3_08 | 0.0150 | *** | 0.0048 | *** | 0.0198 | *** |
| QUARTER_4_08 | -0.0600 | *** | -0.0192 | *** | -0.0793 | *** |
| QUARTER_1_09 | 0.0598 | *** | 0.0192 | *** | 0.0790 | *** |
| QUARTER_2_09 | 0.0389 | *** | 0.0125 | *** | 0.0514 | *** |
| QUARTER_3_09 | 0.0537 | *** | 0.0172 | *** | 0.0709 | *** |
| QUARTER_4_09 | 0.0633 | *** | 0.0203 | *** | 0.0835 | *** |
| QUARTER_1_10 | 0.0541 | *** | 0.0173 | *** | 0.0714 | *** |
| QUARTER_2_10 | 0.0118 | *** | 0.0038 | *** | 0.0156 | *** |
| QUARTER_3_10 | 0.0468 | *** | 0.0150 | *** | 0.0618 | *** |
| QUARTER_4_10 | 0.0767 | *** | 0.0246 | *** | 0.1013 | *** |
| QUARTER_1_11 | 0.0926 | *** | 0.0297 | *** | 0.1223 | *** |
| QUARTER_2_11 | 0.0589 | *** | 0.0189 | *** | 0.0778 | *** |
| QUARTER_3_11 | 0.0441 | *** | 0.0141 | *** | 0.0583 | *** |
| QUARTER_4_11 | 0.0742 | *** | 0.0238 | *** | 0.0979 | *** |
| QUARTER_1_12 | 0.1037 | *** | 0.0332 | *** | 0.1369 | *** |
| QUARTER_2_12 | 0.1078 | *** | 0.0346 | *** | 0.1424 | *** |
| QUARTER_3_12 | 0.1094 | *** | 0.0351 | *** | 0.1445 | *** |
| QUARTER_4_12 | 0.1213 | *** | 0.0389 | *** | 0.1602 | *** |
| QUARTER_1_13 | 0.1531 | *** | 0.0491 | *** | 0.2022 | *** |
| QUARTER_2_13 | 0.1563 | *** | 0.0501 | *** | 0.2065 | *** |
| QUARTER_3_13 | 0.1783 | *** | 0.0571 | *** | 0.2354 | *** |
| QUARTER_4_13 | 0.1790 | *** | 0.0574 | *** | 0.2364 | *** |
| QUARTER_1_14 | 0.1835 | *** | 0.0588 | *** | 0.2423 | *** |
| QUARTER_2_14 | 0.1796 | *** | 0.0576 | *** | 0.2372 | *** |
| QUARTER_3_14 | 0.1946 | *** | 0.0624 | *** | 0.2569 | *** |
| QUARTER_4_14 | 0.1870 | *** | 0.0599 | *** | 0.2469 | *** |
| QUARTER_1_15 | 0.2428 | *** | 0.0778 | *** | 0.3206 | *** |
| QUARTER_2_15 | 0.2222 | *** | 0.0712 | *** | 0.2935 | *** |
| QUARTER_3_15 | 0.2388 | *** | 0.0765 | *** | 0.3153 | *** |
| QUARTER_4_15 | 0.2176 | *** | 0.0697 | *** | 0.2873 | *** |
| LIVING_AREA | -0.0032 | *** | -0.0010 | *** | -0.0042 | *** |
| SINGLE_PLOT_MINUS_LIVING | 0.0002 | *** | 0.0001 | *** | 0.0003 | *** |
| TERRACED_PLOT_MINUS_LIVING | 0.0001 | *** | 0.00003 | *** | 0.0001 | *** |
| NUMBER_OF_ROOMS | 0.0228 | *** | 0.0073 | *** | 0.0302 | *** |
| OWNERSHIP | 0.3136 | *** | 0.1005 | *** | 0.4141 | *** |
| CONSTRUCTION_PERIOD_1500_1650 | 0.0784 | *** | 0.0251 | *** | 0.1035 | *** |
| CONSTRUCTION_PERIOD_1651_1750 | 0.0816 | *** | 0.0261 | *** | 0.1077 | *** |
| CONSTRUCTION_PERIOD_1751_1810 | -0.0021 |  | -0.0007 |  | -0.0028 |  |
| CONSTRUCTION_PERIOD_1811_1900 | -0.0160 | *** | -0.0051 | *** | -0.0211 | *** |
| CONSTRUCTION_PERIOD_1901_1910 | -0.0236 | *** | -0.0076 | *** | -0.0312 | *** |
| CONSTRUCTION_PERIOD_1911_1920 | -0.0133 | ** | -0.0043 | ** | -0.0176 | ** |
| CONSTRUCTION_PERIOD_1921_1930 | -0.0491 | *** | -0.0157 | *** | -0.0648 | *** |
| CONSTRUCTION_PERIOD_1931_1970 | -0.0857 | *** | -0.0275 | *** | -0.1132 | *** |
| CONSTRUCTION_PERIOD_1971_2000 | -0.1917 | *** | -0.0614 | *** | -0.2532 | *** |
| CONSTRUCTION_PERIOD_2001_2010 | -0.0207 | *** | -0.0066 | *** | -0.0274 | *** |
| GEN_REN_AFTER_2010 | 0.1734 | *** | 0.0556 | *** | 0.2290 | *** |
| GEN_REN_BEFORE_2010 | -0.0086 |  | -0.0027 |  | -0.0113 |  |
| ELEVATOR | 0.0093 | *** | 0.0030 | *** | 0.0123 | *** |
| BALCONY | -0.0047 | *** | -0.0015 | *** | -0.0061 | *** |
| CENTRAL_HEATING | -0.0007 |  | -0.0002 |  | -0.0010 |  |
| FLOOR_MINUS_2 | -0.0465 |  | -0.0149 |  | -0.0615 |  |
| FLOOR_MINUS_1 | 0.0264 |  | 0.0085 |  | 0.0349 |  |
| FLOOR_1 | -0.0154 | *** | -0.0050 | *** | -0.0204 | *** |
| FLOOR_2 | 0.0071 | *** | 0.0023 | *** | 0.0094 | *** |
| FLOOR_3 | 0.0215 | *** | 0.0069 | *** | 0.0284 | *** |
| FLOOR_4 | 0.0406 | *** | 0.0130 | *** | 0.0536 | *** |
| FLOOR_5 | 0.0766 | *** | 0.0246 | *** | 0.1012 | *** |
| FLOOR_6 | 0.1110 | *** | 0.0356 | *** | 0.1466 | *** |
| FLOOR_7 | 0.1036 | *** | 0.0332 | *** | 0.1368 | *** |
| FLOOR_8 | 0.1015 | *** | 0.0325 | *** | 0.1340 | *** |
| FLOOR_9 | 0.0770 | *** | 0.0247 | *** | 0.1016 | *** |
| FLOOR_10 | 0.0870 | *** | 0.0279 | *** | 0.1149 | *** |
| FLOOR_11 | 0.1089 | *** | 0.0349 | *** | 0.1438 | *** |
| FLOOR_12 | 0.0926 | *** | 0.0297 | *** | 0.1222 | *** |
| FLOOR_13 | 0.1013 | *** | 0.0325 | *** | 0.1337 | *** |
| FLOOR_14 | 0.1194 | *** | 0.0383 | *** | 0.1577 | *** |
| FLOOR_15 | 0.1771 | *** | 0.0567 | *** | 0.2338 | *** |
| FLOOR_16 | 0.1463 | *** | 0.0469 | *** | 0.1932 | *** |
| FLOOR_17 | 0.2350 | *** | 0.0753 | *** | 0.3103 | *** |
| FLOOR_18 | 0.2892 | *** | 0.0927 | *** | 0.3819 | *** |
| FLOOR_20 | 0.2236 | *** | 0.0717 | *** | 0.2953 | *** |
| FLOOR_21 | 0.2810 | *** | 0.0901 | *** | 0.3710 | *** |
| FLOOR_22 | 0.3821 | *** | 0.1225 | *** | 0.5046 | *** |
| FLOOR_23 | 0.4283 | ** | 0.1373 | ** | 0.5656 | ** |
| FLOOR_24 | 0.2650 | *** | 0.0849 | *** | 0.3499 | *** |
| ln(KINDERGARTEN) | -0.0015 |  | -0.0005 |  | -0.0019 |  |
| ln(SCHOOL) | 0.0007 |  | 0.0002 |  | 0.0010 |  |
| ln(UNIVERSITY) | -0.0167 | *** | -0.0053 | *** | -0.0220 | *** |
| ln(CINEMA) | -0.0439 | *** | -0.0141 | *** | -0.0580 | *** |
| ln(THEATRE) | -0.0557 | *** | -0.0179 | *** | -0.0736 | *** |
| ln(ARTS_CENTER) | -0.1289 | *** | -0.0413 | *** | -0.1702 | *** |
| ln(COMMUNITY_CENTER) | -0.0019 |  | -0.0006 |  | -0.0025 |  |
| ln(SWIMMING_POOL) | 0.0637 | *** | 0.0204 | *** | 0.0841 | *** |
| ln(PENDELSTATION) | 0.0077 | *** | 0.0025 | *** | 0.0101 | *** |
| ln(SUBWAY) | -0.0004 |  | -0.0001 |  | -0.0005 |  |
| ln(CENTRAL_STATION) | -0.0002 |  | 0.0000 |  | -0.0002 |  |
| ln(MULTIFUNCTIONAL_0) | -0.0053 | *** | -0.0017 | *** | -0.0070 | *** |
| ln(MULTIFUNCTIONAL_1) | -0.0077 | *** | -0.0025 | *** | -0.0101 | *** |
| ln(MULTIFUNCTIONAL_2) | -0.0045 | *** | -0.0014 | *** | -0.0059 | *** |
| ln(MULTIFUNCTIONAL_3) | -0.0247 | *** | -0.0079 | *** | -0.0326 | *** |
| ln(MULTIFUNCTIONAL_4) | -0.0165 | *** | -0.0053 | *** | -0.0218 | *** |
| ln(MULTIFUNCTIONAL_5) | -0.0784 | *** | -0.0251 | *** | -0.1036 | *** |
| ln(WATER) | -0.0251 | *** | -0.0080 | *** | -0.0331 | *** |
| GREENERY_BUF_500 | -0.0019 | *** | -0.0006 | *** | -0.0026 | *** |
|  |  |  |  |  |  |  |
| Rho | 0.2503 | *** |  |  |  |  |
| Lambda | 0.5772 | n/a |  |  |  |  |
| Residual variance (sigma squared) | 0.0261 | n/a |  |  |  |  |
| Number of observations | 173052 | n/a |  |  |  |  |

*** - significant at 10% level, ** - significant at 5% level, * - significant at 1% level
